# Evidence for rule versus exemplar learning strategies as stable individual differences independent from working memory

**DOI:** 10.3758/s13421-025-01752-7

**Published:** 2025-08-07

**Authors:** Samuel A. Herzog, Micah B. Goldwater

**Affiliations:** School of Psychology, The University of Sydney, Sydney, NSW 2006 Australia

**Keywords:** Individual differences in concept learning, Exemplar learning, Rule abstraction, Concept learning and transfer

## Abstract

Evidence for two qualitatively different learning strategies has emerged from the function- and category-learning literatures: a *rule-based* and an *exemplar-based* strategy. With a rule-based strategy, learners abstract some common principle from training items, which allows extrapolation to novel instances. With an exemplar-based strategy, learners encode training items without abstraction, which facilitates generalisation based on surface similarity to trained items. Previous studies offer preliminary evidence that strategies are stable; that is, convergent performance was found across pairs of disparate tasks. The current paper advances this work by examining whether performance across a battery of tasks converges, providing evidence for a latent variable underlying learning strategy. Subjects completed five learning strategy and three working memory tasks. Using data reduction and latent structure modelling methods, we found evidence for a general strategy construct that was unrelated to working memory. This is important because it shows that differences in learning strategy are not simply due to differences in working memory.

## Introduction

How do learners differ? It is generally accepted that learners differ from each other in their cognitive capacity, for example, working memory capacity. These differences can be quite stable across contexts, and further often are associated with differences in real-world learning outcomes (e.g., Engle et al., 1999). However, the association between individual differences in working memory or any cognitive capacity with learning outcomes does not entail that learners differ in the processes or strategies they employ. In some applied learning domains, such as in math and literacy education, there has been evidence that differences in working memory capacity lead to differences in learning strategies (e.g., Swanson, 2014), but in areas of laboratory research that focus on formalising learning processes, such as category and associative learning, historically it has been assumed (either implicitly or explicitly) that the same learning process characterises all learners. For example, experiments on category learning are often aimed at discriminating between two competing models of categorisation (such as models that abstract categorisation rules, or models that encode exemplars without abstraction), with the implication that the winning model captures a universal learning process (e.g., Nosofosky, Kruschke, McKinley, 1992, among many others).

When investigating learning process models in these areas, variation in working memory capacity across learners has not typically been the focus. This variation may relate to global task performance, for example, the number of experimental trials it takes to reach a learning criterion. However, this may be most frequently treated as noise in the model-fitting process, rather than a primary target of investigation because global task performance is rarely the measure to distinguish between process models. Instead, these experiments use clever designs that focus on divergent patterns of generalisations tested after learning is complete, which allow for interrogation of what was encoded during learning (e.g., abstract rules that summarize the information from a collection of exemplars vs. direct encoding of each exemplar in the collection).

Complicating the universal-process approach is a recent line of research that examines stability in differences in learning strategies across disparate tasks, that, for example, some learners may be more prone to abstraction, while others may be more prone to store exemplars in memory (McDaniel et al., 2014; however, see Zaman et al., 2023, for another approach). This work’s examination of stability on learning strategy differences is a step towards uniting theoretical research on learning process with research on individual differences in cognitive capacity.

### Mixed evidence for stable individual differences in learning strategy

Recent evidence from function- and category-learning suggests neither of two of the most common classes of models (*rule-based* and *exemplar-based*) characterize a universal process but instead are distinct strategies people engage with consistently across tasks. That is, this evidence suggests that learning strategy and not just cognitive capacity is a stable individual difference.

Prior research establishes these two distinct strategies. McDaniel and colleagues (2014) trained participants to predict output values from input values in a function-learning task. Inputs mapped to outputs via a ‘V-shaped’ function. During training, input values were within a restricted range near the inflection point of the ‘V’, and so all output values during training were relatively low. After training, participants were tested on novel values inside and outside the training range. Learning the rule of the V shape, which produces ever-increasing output values as input values get further from the inflection point, affords extrapolation of higher predicted output values, outside the range of what occurred during training.

All participants could accurately interpolate new values within the range, but only some could extrapolate outside the range (dubbed ‘rule learners’). Thus, using a rule-based strategy, participants abstract a common principle from training items, which allows them to extrapolate to novel instances. To do so, the authors suggested that rule-learners held in mind simultaneously the input and output values of multiple trials to determine how the same rule could produce each pair of values. Conversely, with an exemplar-based strategy, participants encode training items without abstraction, which allows generalisation only based on perceptual or feature-based similarity to stored examples (see ALCOVE model, Kruschke, 1992, or discussion of feature-based generalisation in Shanks & Darby, 1998). The exemplar learners had ‘flat ‘ extrapolation profiles in that they predicted output values within the limit of what they saw during training, even as input values diverged. This strategy does not seem to require the active comparison of multiple trials; instead learning can progress by encoding trials one-at-a-time.

Little and McDaniel (2015) instead used a categorisation task to examine differences in rule- versus exemplar-learning strategies. Rather than learn how to predict numerical output values from input values, wherein every input has a unique output, categorisation tasks present stimuli to be classified into a small number of predetermined categories. Here, Little and McDaniel trained participants on a shape categorisation task where participants learned to categorise bi-shape stimuli into two categories. Without any explicit instruction on what strategy to use, participants trained to criterion either by memorising the exemplars or by learning the rule which concerned relationships between the two component shapes. During transfer, newly introduced ambiguous items elicited two kinds of response: subjects either correctly classified new items based on the relational rule (rule learners), or they classified new items by perceptual similarity to the training items (exemplar learners). Likewise in a second task (without any hints of what strategy to use), wherein participants learned categories of letter strings, they either memorized the strings or realised that the final letter of a string corresponded to category membership (see Little & McDaniel, 2015, study 2). As with shape categorisation, participants either systematically generalised based on the rule, or categorised new letter strings based on perceptual similarity to stored examples. Importantly, this paper showed that their generalisation profiles matched their self-reports of learning a rule or memorising exemplars, suggesting these strategy choices are intentional, or at least that learners are self-aware.

Despite the above research, there is still insufficient evidence on whether learning strategy differences are stable. McDaniel et al. (2014) were first to consider this – would participants classified as rule learners in a function-learning task tend to indicate rule discovery in a new task? Indeed, they found that strategy in the function-learning task persisted to a new conceptual task. Goldwater et al. (2018) extended this finding and further corroborated the suggestion from McDaniel et al. that central to rule-learning strategies is the active comparison of stimuli across trials.

Goldwater et al. (2018) developed a perceptual category learning paradigm wherein the categories could be learned via superficial features (i.e., the colour pattern in the stimuli) or a spatial relational rule among the stimulus elements, similar to the two distinct ways to categorize the bi-shape stimuli in the Little and McDaniel shape task. This work also used the letter-string task from Little and McDaniel to identify rule or exemplar strategies in an independent task. In the perceptual category learning task, learning sequence was manipulated. Half of the participants had a blocked learning sequence wherein there was a 75% chance that consecutive trials would be from the same category, and the other half had an interleaved learning sequence wherein consecutive trials had a 25% chance of sharing category membership. This manipulation had no effect for exemplar learners but had a large effect for rule learners. Rule learners learned the relational rule in the blocked condition where consecutive trials supported comparison of exemplars that shared a relational rule. The rule learners, however, focussed on the features in the interleaved condition where that within-category comparison was supported more rarely. On the other hand, the exemplar learners learned the featural aspect, and not the relational rule of the categories regardless of the learning sequence condition. This interaction of learning sequence and learning strategy suggests that rule learners are trying to compare across trials, but exemplar learners are not. If the learner is not comparing across trials, then the sequence of trials makes no difference.

Further corroborating the stability of these strategies is research connecting them to real educational environments. McDaniel and colleagues (McDaniel, Cahill, Frey, Limeri, & Lemons, 2022; McDaniel et al., 2018) identified rule and exemplar learners using the function learning task and examined relationships with their university course performance in biology and chemistry, respectively. Exemplar and rule learners performed equally well in exam questions that directly tested material the students had previously seen, but rule learners did better on transfer questions that tested if they could apply their knowledge to novel materials. Goldwater et al. (2018) connected learning the relational rule in the perceptual categorisation tasks to self-reports of using ‘elaborative’ learning strategies in their university coursework such as comparing their lecture material to their reading. Triangulating these findings, Zepeda and Nokes-Malach (2021) showed that self-reports of comparison in coursework was positively correlated with university exam performance.

Putting this all together, people who: (1) actively compare across trials in laboratory tasks, (2) actively compare materials in their coursework, are (3) more likely to learn abstract rules that support extrapolation in lab tasks and are (4) more likely to be able to transfer their learning to novel questions on university exams. This certainly suggests stability in learning strategies across contexts.

Further, the connections between individual differences in rule learning, transfer, and comparison is consistent with experimental work on analogical comparison. Instructions and task designs to scaffold comparison (1) highlight the common relations among the comparates, which (2) supports the abstraction of those underlying relations, and (3) fosters knowledge transfer of the relational abstraction to novel instances (Gentner, 2010; Gick & Holyoak, 1983). Scaffolding comparison supports learning and transfer in both lab-based relational categorisation tasks (Doumas & Hummel, 2013; Kurtz, Boukrina, & Gentner, 2013) and formal education settings (Jacobson et al., 2020; Goldwater and Schalk 2016; Rittle-Johnson & Star, 2007). Most relevant, perhaps, is that analogical comparison is arguably critical to rule learning across areas of cognition (Gentner & Medina, 1998).

However, because this experimental research points to how task prompts to compare can support rule learning and transfer, this suggests that differences between rule and exemplar learners are perhaps not so entrenched. Indeed, there is extensive research that people use a combination of rules and exemplars in category learning, with emphasis shifting based on conditions such as the size of the training set (Thibaut et al., 2018). Generalisation to novel instances in both lab tasks and real-world medical settings has been shown to be based on both rules and exemplar similarity at once (Brooks et al., 1991; Hahn et al., 2010). Further, in more detailed analyses of individual differences, some people may start out a task as a rule or exemplar learner, but then depending on how well they see their strategy working, they may switch (Gouravajhala et al., 2020). Gouravajhala et al. (2020) suggest that exemplar and rule strategies are perhaps ‘focal orientations’, but both strategies are available to all people.

On the one hand, experimental research shows that both kinds of strategies are available to learners in laboratory tasks. On the other hand, the connections between the lab tasks and real-world education results suggest that even if both kinds of strategies are available, the bias for any individual towards one or the other may be quite strong when they are left to their own devices, such as when studying for final exams.

The current research examined the strength and stability of these focal orientations towards rule or exemplar strategies. Our approach was to examine the consistency of these learning strategies (without experimental manipulation) using the statistical methods of individual differences research to identify stable traits, such as in personality and intelligence assessment. The above discussed research on learning strategy only examined connections between two tasks or one task connected to education performance, in any given study. Instead, we use data reduction and latent structure modelling across a broad array of tasks, standard methods of individual difference research. The assumption here, in assessment research for example, is that predicting performance on any given test, which is observable, is best based on some underlying variable (such as cognitive ability), which is not directly observable. To best account for the variables driving performance, data reduction and latent structure modelling capture the interrelationships among a battery of tasks, if they exist (Hambleton & Cook, 1977). Just a pair of tasks is insufficient for reliable data reduction and evaluating whether a key construct, such as a learning strategy, is driven by an underlying latent variable.

To be clear, given the experimental research mentioned above, we are not suggesting these learning strategies are traits in the same sense as personality and intelligence research. Simple instructional prompts most likely do not flip intelligence test scores as they can seemingly flip learning strategies. However, the same statistical methods can be used to better understand the nature and consistency across tasks of these focal orientations.

Critically, these data reduction and modelling approaches can reveal more than just stability; this approach allows for deeper insight into the nature of the underlying construct by, for example, examining variations in the factor or component loading of different tasks (i.e., how related each task is to the key underlying construct). Perhaps most critically, these methods can discriminate between distinct traits, for example, between learning strategy and working memory capacity, if performance across the task set can be reduced into two coherent and distinct dimensions of shared performance.

The current research had two primary questions concerning the existence of a consistent focus towards rule or exemplar-based learning strategies. First, could we find evidence for a latent factor distinguishing rule from exemplar learning strategies across several tasks? A battery of tasks sufficiently converging to provide evidence for a latent variable is far from guaranteed. Second, if we did find this evidence, was this latent factor distinct from working memory capacity? This second question is important for answering whether these cognitive strategies can be distinguished from cognitive capacities. Many tasks confound these two factors. For example, performance on the Cognitive Reflection Task is based on cognitive capacity but also preferred cognitive styles and thinking dispositions (e.g., Frederick, 2005; Toplak et al., 2014).

Because rule learning requires holding multiple stimulus elements in mind at once, it has been hypothesized that rule-learning strategies would be supported by greater working-memory capacity. There is currently mixed evidence if working memory is linked to learning strategy, and the current research aimed to resolve this issue (see below).

Highlighting the strength of a data reduction and latent structure modelling approach, two sets of tasks can be correlated, but still show distinct principal components or latent factors. Famously, intelligence has this structure wherein, for example, verbal and visual intelligence are both correlated and distinct (e.g., Canivez et al., 2016). By using multiple rule-versus-exemplar tasks, and multiple working memory tasks, this research is able to discover this kind of pattern.

### Mixed evidence for a relationship between working memory capacity and learning strategy

Working memory is the number of elements in memory that can be actively related to each other simultaneously (see Halford, Wilson, & Phillips, 1998). It is a “multicomponent system responsible for active maintenance of information in the face of ongoing processing and/or distraction” (Conway et al., 2005, p. 770). To abstract-away the features or common relational structure that govern category membership in a category-learning task, or discover the underlying function generating data, one must simultaneously hold in mind multiple category exemplars or data points. Thus, it is feasible that this process could be facilitated by greater working memory, as someone with high working memory can consider more elements simultaneously. Indeed, McDaniel et al. (2014) found greater working memory in rule versus exemplar learners in the function-learning task: “Greater working memory capacity […] allow[s] […] maintain[ing] several cue-criterion trials in mind […] to abstract the function rule” (McDaniel et al., p. 674).

Wills, Barrasin and McLaren (2011) found a similar connection between strategy and working memory using a ‘patterning’ task, where participants learned how single and pairs of cues predicted an outcome. When individual cues predicted the same outcome, pairing those cues together reversed the outcome. For example, if eating eggs and broccoli on their own predicted a healthy outcome, then eating them together elicited an allergic reaction (we note the relationships in patterning tasks do not reflect real-world associations). This was a relational rule because it is based on the relationship between cues (predicting the same outcome), regardless of the identity or features of each cue. Low working memory individuals used the memory of single cues and their individual outcomes to predict the result of new pairs of familiar cues. But high working memory individuals used the rule they had discovered that related how the pattern of single cues predicted the effect of pairing cues together. Thus, it is possible that exemplar learners are not rule learners simply because they lack the working memory capacity required to learn complex rules. This would suggest that strategy differences are not just choices but are rooted in cognitive capacity differences.

There is recent related research that further suggests a connection between relational rule discovery and working memory capacity. Gray and Holyoak (2020) used a larger battery of tasks than the studies discussed above (such as McDaniel et al., 2014, or Goldwater et al., 2018), and focussed on reasoning and problem-solving, rather than learning and memory (which are two distinct literatures in cognitive psychology). In the rule-versus-exemplar learning tasks, knowledge of categories, functions, or associative patterns is built across dozens of trials. This contrasts with the reasoning and problem-solving tasks wherein the content of each problem or trial are not particularly contingent on each other (besides, e.g., increasing levels of difficulty). However, this work is relevant because it specifically examines relational reasoning (i.e., reasoning with the extrinsic relations among things, such as in reasoning via analogy), and again rule-learning requires the discovery and generalisation of common relations among stimulus elements across trials.

Importantly, Gray and Holyoak (2020) showed consistent individual differences in relational skill across several relational reasoning and problem-solving tasks (using a composite score, rather than latent variable methods), and further that working memory capacity was correlated with their composite relational reasoning score. Included in their battery is Raven’s Progressive Matrices (RPM; Raven, 2000), which are a standard measure of fluid intelligence, and requires discovering a relational rule among novel complex shapes in every trial. RPM and working memory is well known to be highly correlated, and some definitions of working memory even focus on its role in relational binding (Chuderski, 2013, 2014; Halford, Wilson, & Phillips, 1998).

However, the contrast between the methods of Gray and Holyoak with the current research points to why we may not expect the same strong relationship between working memory and a rule-based learning strategy and between working memory with relational reasoning skill. Critical to tasks that distinguish rule- and exemplar-learning strategies is that either strategy can lead to ceiling performance during the learning phase, so there is no obvious task pressure to use one strategy or another. The two strategies diverge on generalisation trials after an extended learning phase. In contrast, tasks like RPM (Raven, 2000), have a single correct answer that require relational rule discovery, hence the characterisation that Gray and Holyoak’s task battery is assessing ‘skill’ rather than ‘strategy’. When every problem is unique, an exemplar-memory approach does not apply. Goldwater et al. (2018) showed that RPM performance was correlated with overall accuracy during the learning and test phases of a category-learning task but did not differentially predict relational rule-learning versus exemplar-based strategies. This gives evidence that the ability to discover relational rules does not necessarily mean a rule-learning strategy will be deployed in learning tasks with other apt options available.

The study by Goldwater et al. (2018) further adds to the inconsistent evidence of a connection between working memory and relational rule-discovery because it finds the same kind of pattern with working memory as it did for RPM: working memory was predictive of overall task performance but did not distinguish rule learning from exemplar strategy use.

This pattern from Goldwater et al. (2018) is similar to earlier work by Lewandowsky and colleagues. They found evidence that working memory capacity was correlated with overall categorisation performance regardless of the kind of categories to be learned (Lewandowsky, Yang, Newell, & Kalish, 2012), or which categorisation strategy was used (Craig & Lewandowsky, 2012), either focusing on just a subset of feature-dimensions or encoding entire exemplars. In addition, Little and McDaniel (2015) did not find differences in working memory between rule and exemplar learners using shape and letter-string categorisation. This could be because here learning strategy was assessed by participants’ self-report, rather than transfer performance, as in McDaniel et al. (2014) and Wills et al. (2011), but Goldwater et al. (2018) used both self-report and association with another categorisation task and found no strategy-working memory connection.

Thus, there are (at least) two papers that report a connection between working memory and rule-learning strategy, plus the widespread evidence that relational skill is associated with working memory, but there are (at least) three papers that show no connection between learning strategy and working memory in category-learning tasks. In addition, both an anonymous reviewer and audience members at an oral presentation of this research suggested reasons to think that higher working memory capacity may be associated with exemplar strategies rather than rule because of the working memory it takes to commit exemplars to long-term memory. In sum, the evidence for this relationship is mixed.

### The current research

Here we used eight tasks to establish the convergent and discriminant validity of rule-versus-exemplar measures: five for learning strategy and three for working memory. This allows us to examine if the inconsistency of evidence between working memory and learning strategy is due to the two being correlated despite distinct underlying factors. Four of the five strategy measures have been discussed above. They were the function-learning (McDaniel et al., 2014), both the shape and letter-string categorisation tasks (Little & McDaniel, 2015), and patterning (Shanks & Darby, 1998) tasks. See the *Methods* section below for full details.

The fifth strategy task is distinct from the other four in that it does not measure learning and generalisation. In the ‘picture-mapping’ measure from Markman and Gentner (1993), subjects are asked to find object correspondence in pairs of scenes showing similar semantic relationships (e.g., two scenes of food sharing). Subjects can either match objects based on similarity in perceptual features (e.g., matching a person to a similar looking person), or by abstract relational role (matching a ‘food giver’ to a ‘food giver’). Thus, picture mapping differentiates subjects who spontaneously look for an underlying relationship versus those who use surface similarity.

Above, we explained the connection between relational learning and rule learning on the one hand, and exemplar learning and attention to features on the other. Including the picture-mapping task empirically tests this connection more explicitly because this task was developed to differentiate what factors (e.g., scaffolding comparison) support an attention to extrinsic relations between objects or the intrinsic features of objects. In addition, we note that the inclusion of the picture-mapping tasks allows the current approach to be bridged with Gray and Holyoak (2020), who included this picture-mapping task in their relational reasoning battery. This task is appropriate for both projects because unlike RPM, which only has correct relational-rule answers, there are clear non-relational answers in the picture-mapping task (the perceptual matches). We note the picture-mapping task was highly correlated with their relational composite score (*r* = 0.67 in Experiment 1; *r* = 0.59 in Experiment 2).

The five tasks were selected because they come from distinct areas within the learning and cognition literature – function learning, category learning, associative learning, and analogy, respectively. Despite the distinction in the task designs and underlying cognitive processes across these areas, they all share the distinction between learning and thinking with abstractions based on shared structural relations and learning and thinking with the more superficial intrinsic features of stimulus elements.

To measure a working memory factor, we included the commonly used working memory tasks Operation span, Reading span, and Symmetry span (henceforth referred to as O-span, R-span, and S-span, respectively). Previous research suggests strong correlations between these measures (*r* = 0.55–0.73; Kane et al., 2004).

In sum, if the score variance from the five rule-versus-exemplar tasks can be explained by a latent variable, this provides valuable evidence that strategy differences are stable.

### Predictions

We expected performance from the five rule-versus-exemplar tasks to converge, providing evidence for a latent variable. We also expected working memory task scores to converge but diverge from the rule-versus-exemplar tasks. That is, correlations for rule-versus-exemplar with the other rule-versus-exemplar tasks (and working memory with the other working memory tasks) to be higher than those between the rule-versus-exemplar tasks with the working memory tasks. That is, our first hypothesis was that a principal component analysis (PCA), which identifies patterns of shared variance across task performance, would return two components – one for learning strategy, and a second for working memory. To test whether a two-factor latent structure could account for these observed patterns, we followed the PCA with confirmatory factor analysis (CFA). Thus, our second hypothesis was that CFA would show superior fit for a two-factor model aligned with the structure of the PCA, in comparison to models with one or zero latent factors.

If this two-step process, data reduction (via PCA) and latent structural modelling (via CFA), reveals consistent evidence for two underlying constructs, this would establish both convergent and divergent validity of the learning strategy construct, precluding the possibility that rule-versus-exemplar task performance was converging due to an extraneous factor like motivation. But even with this pattern of results, it would still be possible to have a significant positive correlation between working memory capacity and rule strategy. Given the mixed evidence of this connection in the past, this was a plausible outcome. Thus, our third hypothesis was that there would still be some positive correlation between learning strategy and working memory, despite them loading on distinct components.

## Method

### Participants

Ninety-six participants were recruited through Amazon’s Mechanical Turk (https://requester.mturk.com). These data were collected in 2017. We based our sample size on the simulation studies of Mundfrom et al. (2005) where they investigated the necessary sample size to reliably recover the latent variable structure of a population under different conditions. The current research aimed to be able to recover two factors from eight variables (a 4:1 variable to factor ratio) assuming a wide range of communalities across the variables. Mundfrom et al. (2005) recommended a sample size of N = 90 for these conditions. We used an online sample because we were concerned that an elite university sample would not have adequate range of cognitive capacities.

Participants tested on own computers and were paid $US 8 per hour. Tasks were broken down into three 40-min blocks and one 30-min block and were counterbalanced within and across blocks (for a total of 150 min). Participants had 48 h to complete all blocks and were encouraged to break in-between. They were instructed not to write anything while testing.

### Rule- versus exemplar-strategy tasks

#### Function-learning task

**Fig. 1:**
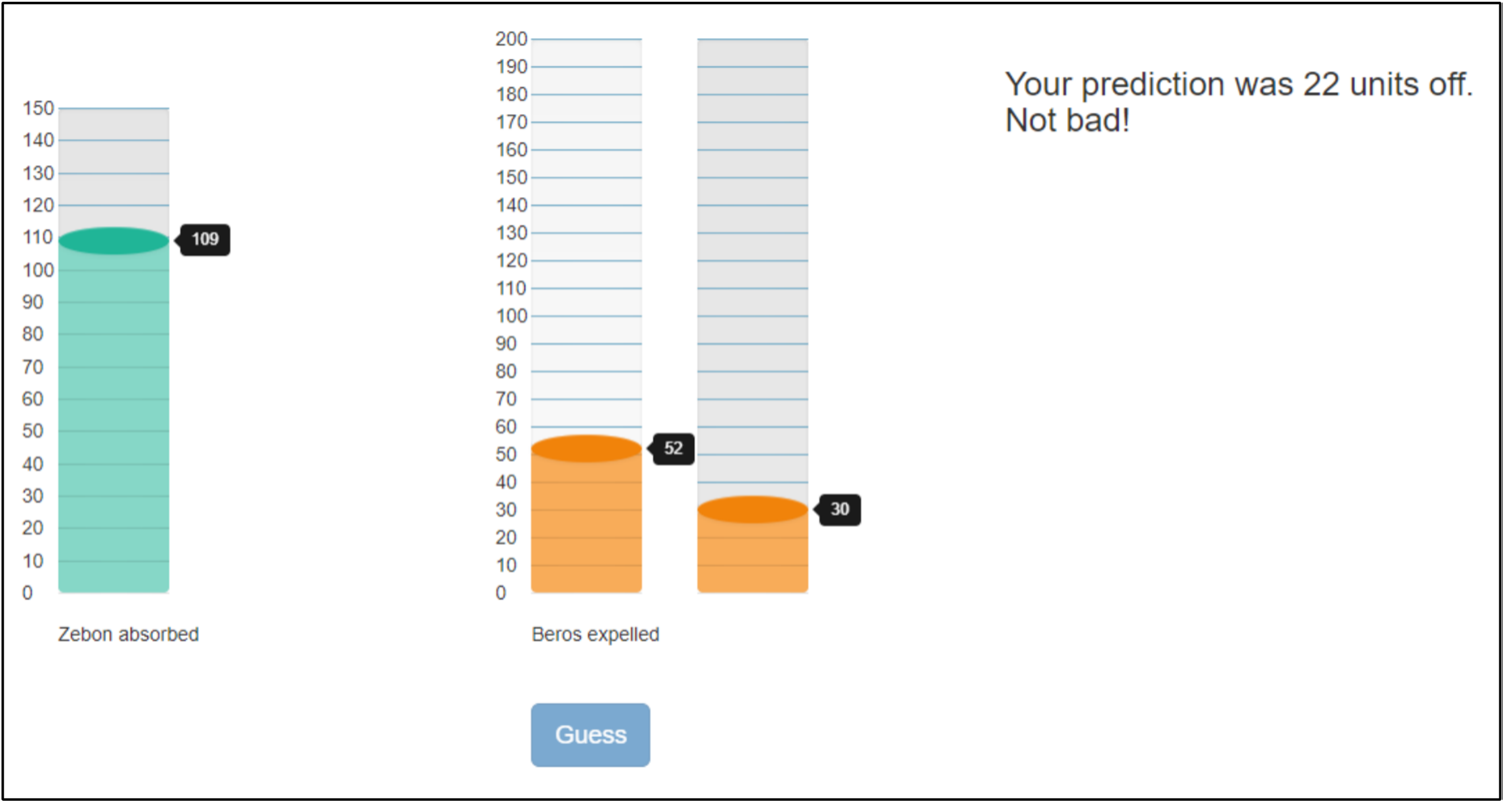
For function-learning, participants saw Zebon absorbed on the left. They then used the mouse to select how much Beros they expected to be expelled. After, they saw the actual Beros expelled plus received corrective feedback, e.g. e.g., ‘Your prediction was 22 units off. Not bad!’

As per McDaniel et al. (2014), function-learning had a training and transfer phase. Before training, participants had to imagine they worked for NASA and were investigating a newly discovered Mars organism. The organism absorbed some amount of a fictional element ‘Zebon’, and participants had to predict how much ‘Beros’ would be released. There was a vertical bar displaying the amount of Zebon and participants estimated how much Beros they expected to be released (see Fig. 1). Participants were given feedback on each trial as determined by the following formula: for inputs < 100, Beros expelled = 229.2 – (2.197*Zebon); for inputs > 100, Beros expelled = –210 + (2.197*Zebon). This created a bi-linear V-shaped function, where 100 is the inflection point where the linear function swaps between negative and positive slopes, hence the V-shape. Participants received error feedback accordingly, calculated by the difference between their output and the value the function produced: when error was < 5, ‘Great job!’; < 15, ‘Good job!’; < 30, ‘Not bad!’; ≥ 30, ‘Try harder!’. There were ten training blocks of 20 randomised trials, with each trial using an odd number between 80 and 120 for the amount of Zebon. After each block, participants’ average error for the block was displayed.

Transfer was identical to training but with two exceptions. First, no corrective feedback was provided. Second, two novel input types were tested: 15 interpolated values (randomly selected even numbers between 80 and 120), plus 34 extrapolated values (randomly selected numbers between 50 and 75 and 125 and 150). All participants received the same interpolated and extrapolated values in a pseudo-randomised order.

#### Shape categorisation task

This task was based on Little and McDaniel (2015, study 1). Participants learned to classify shape pairs into two categories. ‘Blickets’ shared the same form or colour for both shapes. ‘Daxes’ did not have the same shape form or colour. Key-presses indicated responses. Stimuli were presented individually, and feedback was given after each trial (e.g., *Correct, this is a blicket*). There were six training blocks of 12 randomly ordered trials.

During transfer, eight new items (four *unambiguous* and four *ambiguous*) plus four training items were tested without corrective feedback. Twelve *novel* transfer items were also tested. Unambiguous items were perceptually similar to trained items and could either be correctly classified based on this or on the rule governing category membership. Ambiguous items were also perceptually similar to trained items, yet only the rule correctly categorised them. Novel items could be correctly classified by the rule, but were not perceptually similar to trained items (see Appendix I for list of original and *novel* items.) Transfer stimuli were administered pseudo-randomly with the same order for all participants.

#### Letter-string categorisation task

This task was based on Little and McDaniel (2015, study 2). Participants learned to classify 12 articulable letter-strings (e.g., ‘lonsink’) into two categories. Letter-strings ending in ‘t’ were plants. Letter-strings ending in ‘k’ were animals. Letter-strings were presented individually and feedback was given after each trial (e.g., *Correct, this is a plant*).

During transfer, 12 new items (four *rule-favoured*, four *exemplar-favoured*, and four *ambiguous*) plus four training items were tested without corrective feedback. Rule-favoured items had novel stems with a ‘-t’ or ‘-k’ ending and could be correctly classified according to the rule. Exemplar-favoured items had the stem of a trained letter-string, but the last letter was now ‘g’. These items could be classified according to perceptual similarity to trained items. Ambiguous items had a trained stem, but ‘-t’ or ‘-k’ endings were swapped (e.g., ‘lonsink’ became ‘lonsint’). (See Appendix I for examples of *rule-favoured*, *exemplar-favoured*, and *ambiguous* items.) Following other previous tasks using this paradigm, ambiguous transfer items were critical in that they led exemplar and rule learners to diverge in their responses; rule learners could correctly classify these stimuli, whereas exemplar learners would demonstrate a propensity for incorrect classification, due to categorisation according to perceptual similarity to memorised exemplars. Transfer stimuli were administered pseudo-randomly with the same order for all participants.

#### Patterning task

**Fig. 2:**
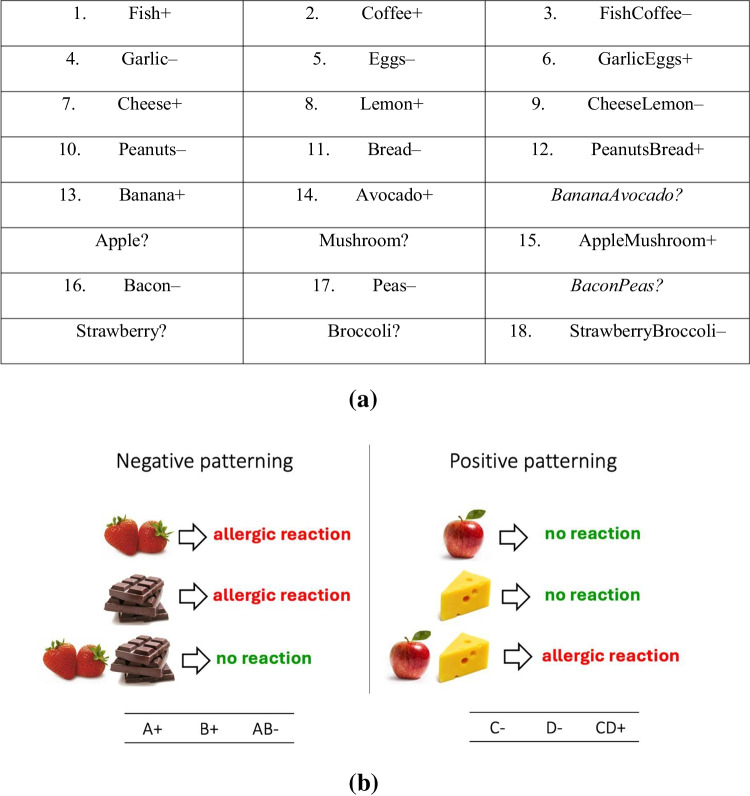
**a** Single and compound cues used in the patterning task (stimuli are written as words here but were images in the task). ‘ + ’ indicate cues that led to a reaction and ‘–’ cues led to no reaction. ‘?’ indicate critical cues only seen in the transfer phase. Italics are used for compound cues only seen in the transfer phase that are the basis of the rule-learning score. **b** Illustrating patterning with example visual stimuli

In this task, participants learned to predict whether single or combinations of foods led to an allergic reaction. During training, various food stimuli were presented individually (e.g., fish, coffee) or as a compound (e.g., fish + coffee). Participants indicated with buttons labelled *ALLERGIC REACTION* and *no reaction* whether they thought the food or combination of foods led to an allergic reaction. Corrective feedback was given. Importantly, whether or not a food or foods led to a reaction was governed by a ‘patterning’ rule; that is, if foods A and B caused a reaction, then A + B did not. The opposite was also true: if foods C and D did not cause a reaction, then C + D did (see Fig. 2 for list of cues).

Eighteen cues (individual and compound) were presented in training. Each individual cue was presented twice per block, and for compounds, both orders (i.e., A + B and B + A) were presented once. Thus, there were 36 trials per block. Training was terminated either when participants reached 32/36 correct responses in a block or else after completing six blocks. During training and transfer, trials were presented randomly.

During transfer, there were 18 previously seen training stimuli plus six critical new cues; for example, Banana and Avocado were seen during training, but Banana + Avocado was only seen during transfer (see Fig. 2). Participants rated from 1 to 100 how likely they thought each cue would be in causing a reaction with no feedback given. The stimuli were presented twice each (single cues twice and both orders of a compound). Critical cues distinguish rule learners from exemplar learners; for example, exemplar learners, who have not discovered the rule but who have memorised the cue-outcome pairings for A and B usually rate AB? as highly likely to cause a reaction; conversely, rule learners, rate AB? as highly unlikely.

#### Picture-mapping task

**Fig. 3:**
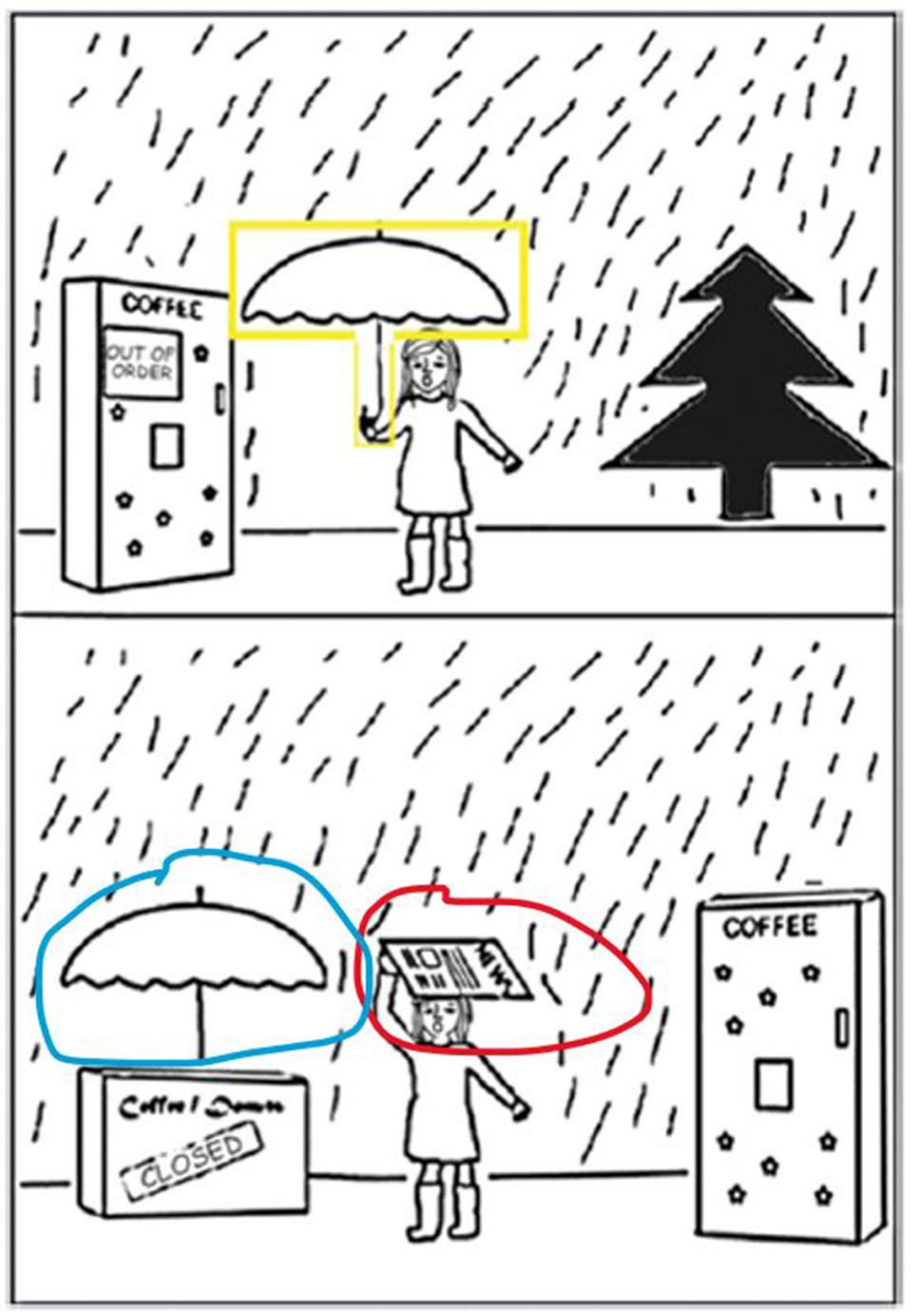
Example item from the picture-mapping task. The yellow box around the target object was shown to the subjects. The blue circle indicates the object-match, and the red circle indicates the relational-match. The blue and red circles were not visible to subjects, are added here for clarification

We used picture-mapping from Markman and Gentner (1993) with extra items added by Tohill and Holyoak (2000). Participants were presented with ten scene pairs and asked to select the item from the lower scene that ‘goes with’ the highlighted object from the upper scene. Two main responses are possible: with a *featural* match, subjects select the same item as highlighted in the upper scene; with a *relational* match, subjects select the object that fulfils the same relational role (see Fig. 3).

### Working memory tasks

We used automated versions of O-span, R-span, and S-span downloaded from the Inquisit task library (http://www.millisecond.com/download/library). In O-span, participants validate a math problem (e.g., (8*2) – 8 = *?*) and then are shown a letter. After three to seven iterations, subjects must recall as many letters as possible in the correct sequence. The letter-sequence lengths from three to seven were tested three times each for a total of 15 trials. For example:

$$\begin{array}{c}({8}^{*}2)-8\hspace{0.17em}=\hspace{0.17em}?)\dots \text{ B}\\ ({4}^{*}6)-3\hspace{0.17em}=\hspace{0.17em}?)\dots \text{ K}\\ ({3}^{*}7)+2\hspace{0.17em}=\hspace{0.17em}?)\dots \text{ D}\end{array}$$

Then the subject would be asked to recall the three letters in order.

R-span is the same as O-span, but instead of math problems, participants must validate whether sentences (e.g., *Most people agree that Monday is the worst stick of the week*) make sense. Letter-sequence lengths three to seven were tested three times for a total of 15 trials.

**Fig. 4:**
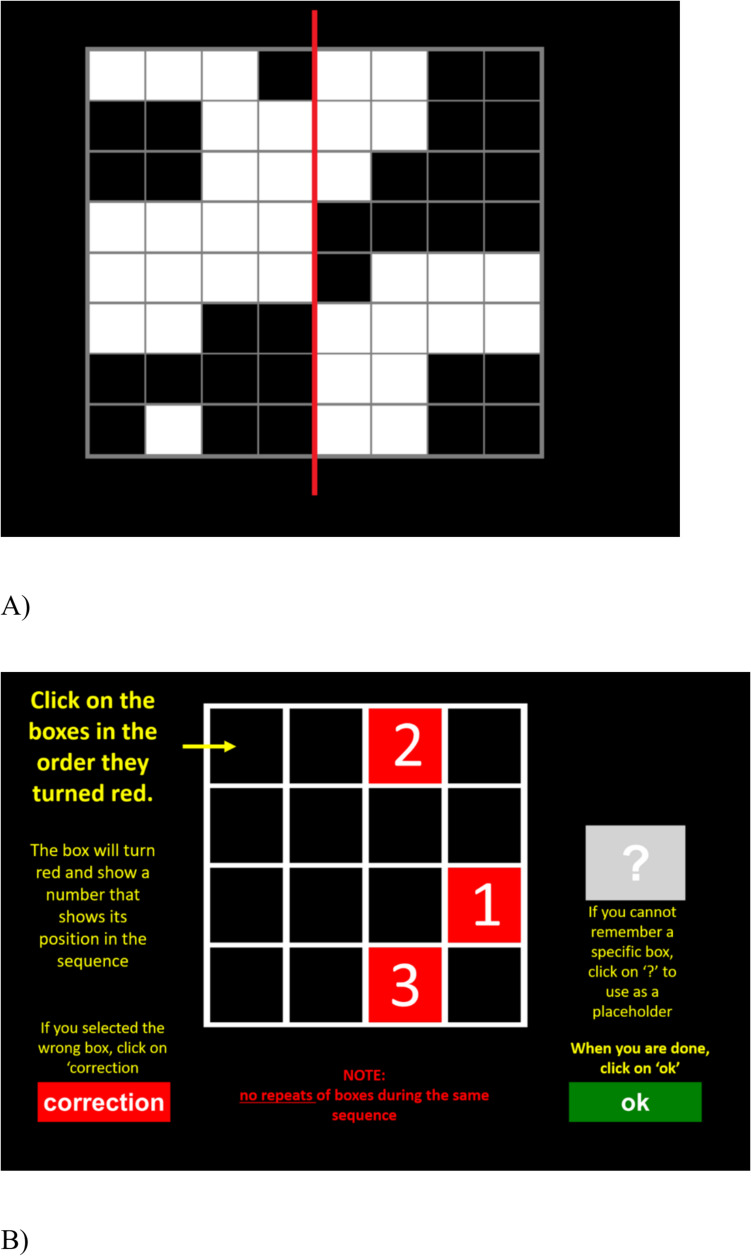
Examples from the symmetry span instruction screens. (**A**) A grid from which to judge symmetry. (**B**) An explanation of the spatial memory test

S-span is similar as O-span and R-span, but here participants must validate whether a pattern on an 8 × 8 grid is symmetrical. Also, instead of letters, subjects memorise the sequence that highlighted squares on a 4 × 4 grid appeared on the screen. There were three repetitions of set sizes two to five for 12 trials total. See Fig. 4.

### Self-reported learning strategy and rule explication

In addition to the rule-versus-exemplar tasks, we included learning strategy self-report measures for shape and letter-string categorisation, and the patterning task. Participants were asked, for example, *In the task where you classified shapes as blickets or daxes, how did you learn the correct way to classify these pictures?* Participants indicated their strategy on a 7-point Likert scale, where 1 = *I was trying to learn the individual items*, and 7 = *I was trying to develop a rule for why items were a member of each category*. Participants were also probed about rule discovery, for example, *If you developed a rule for classifying shapes as blickets or daxes, please explain it below. If you were not able to find a perfect rule for classification, but tried to do so, what was the best rule that you came up with?* Self-reported strategy and rule discovery were elicited after all rule-versus-exemplar tasks were completed. This ensured participants did not overtly reflect on their strategy after a task, influencing strategy on later tasks.

#### ‘Pure strategy’ self-report

Part-way through data collection, we noticed that select participants reported on the rule discovery probe that they had tried to find a rule but were not able to. For example:

1. *I initially tried to see if there was a rule or pattern by looking at the words […] – I did not, however, notice anything pattern wise to judge by so I just tried to learn the individual items.*
2. *I wasn’t sure how to classify [the words] because I always found ‘exceptions’ to my rules like symmetry in the words being animals, or vowels in the same spot on each syllable of the word being an animal, *etc*.*

These participants reported relying on memorisation after failing to find a rule. This raised the possibility that the current method (as per Little & McDaniel, 2015) did not distinguish between a subject’s general orientation to find a rule and their ability to do so. A learner could also plausibly discover a rule accidentally despite relying on memorisation. Thus, we created one additional multiple-choice question that participants were asked alongside each Likert self-report. This was: *In this same task*, *which of these strategies do you think most resembled what you were doing? (1) I was trying to find a rule, and did find a rule, (2) I was trying to find a rule, but did not find a rule, (3) I wasn’t trying to find a rule, and didn’t find a rule, (4) I wasn’t trying to find a rule, but accidentally discovered one, (5) Not sure/none of these.* We thus attempted to capture what we will loosely refer to as ‘pure strategy’; that is, the learner’s general orientation to find a rule or memorise exemplars irrespective of failing to find the rule or accidentally discovering it. We aimed to analyse these new data alongside the usual Likert self-reported strategy to assess any differences.

### Data analysis

Data were analysed in Microsoft Excel (https://www.microsoft.com/), SPSS statistics version 21 (IBM, 2012), FACTOR (http://psico.fcep.urv.es/utilitats/factor/index.html), and the lavaan (Rosseel, 2012), MASS (Ripley, 2002), psych (Revelle, 2024), dplyr (Wickham, François, Henry, & Müller 2023), and ggplot2 (Wickham, 2016) packages for the statistical computing language R.

### Ethics

Ethics approval was obtained from the University of Sydney’s Human Research Ethics Committee (protocol number 2013/1077).

## Results

To evaluate our three hypotheses, we conducted analyses in multiple steps. First, we present how each of the eight tasks were scored and characterize performance on each task. Second, we used PCA to explore the structure of the data and identify potential latent constructs. Third, based on the component solution suggested by PCA, we specified a CFA model to test whether a two-factor latent structure accounted for the observed covariation. After the CFA, we delve into self-report data on learning strategy to gain further insights into the relationships between the intentions of the learners and their performance outcomes.

Hypothesis 1 is that performance on these eight tasks will be best accounted for by a two-component solution, where learning strategy and working memory will load onto separate components. Hypothesis 2 is that the CFA will show that a two-factor structure aligned with the results of the PCA will better fit the covariation data than a one- or zero-factor model. Hypothesis 3 is that despite the support for two latent factors, there will still be some positive correlation between learning strategy and working memory.

### Score calculations

#### Function-learning

Mean error scores were calculated for participants’ final training block, interpolation, and extrapolation trials. This was calculated based on the V-shaped function as explained in the methods section. That is for every trial, subjects made a prediction of the output based on the input. The difference between their predicted output and the function’s output was the error. Only subjects with final training block errors ≤ 11 were included in analysis. Subjects were classified as rule learners or exemplar learners using the method of McDaniel et al. (2014). Participants were classified as V-shape rule learners if the 95% confidence interval around their mean extrapolation trial error was < 34.72. That is, for the extrapolation trials their mean and 95% confidence interval of the errors were calculated as explained above.

Because some subjects are known to extrapolate according to a ‘sine function’ rather than the normal V-shaped function (McDaniel et al., 2014), participants’ mean extrapolation errors were also calculated with respect to this function. It is not a true sine function, but multiple alternations between linear functions with a positive slope and a negative slope. To explain: during the training trials, input values ranged between 80 and 120. From 80–100, the slope was negative, and from 100–120, the slope was positive (making a ‘V’). During extrapolation trials, the inputs ranged from 50–75 and 125–150. The training data from 80–120 are of course consistent with a V-shape, where the data from 50–75 are produced by the same negative slope function as 80–100, and 125–150 are produced by the same positive slope as from 100–120, respectively. This leads to all extrapolation output predictions beyond the training range. However, the training data are also consistent with an alternative function wherein every 20 inputs values, the direction of the slope flips. That is 40–60 is negative, 60–80 is positive, 80–100 is negative, 100–120 is positive, and 120–140 is negative, and 140–160 is positive again. Here extrapolation predictions are not beyond the training range, but a clear rule was still abstracted, and so classifying this generalisation profile as exemplar-based would be inaccurate.

Here, we altered the V-shape function to reflect this alternation and calculated error-scores. Following McDaniel et al. (2014), if the upper limit of a participant’s mean error confidence interval was < 24.09 they were considered a sine learner. Exemplar learners were those who did not show evidence of learning either the V-shape or the sine function. See Table 1 for data exclusion and numbers of rule, sine, and exemplar learners. Mean error scores and standard deviations for subjects’ final training block and interpolation trials are listed in Appendix Table 9.

**Table 1:** Data exclusion, frequencies, means, and SDs for the five learning strategy and three working memory tasks

Task	No. of subjects with data missing	No. of subjects excluded for not reaching criterion	Final no. of subjects used in analysis	Frequency/mean	SD
Function-learning	1	24	71	28 rule learners (16 regular and 12 sine), 43 exemplar learners	n/a
Shape categorisation	4	10	82	36 rule, 46 exemplar	n/a
Letter-string categorisation	4	12	80	20 rule, 60 exemplar	n/a
Patterning	1	9	86	32 rule, 54 exemplar	n/a
Picture-mapping	0	0	96	4.27	2.74
O-span	0	4	92	48.48	19.46
R-span	5	4	87	48.13	19.61
S-span	3	13	80	21.44	10.46

**Fig. 5:**
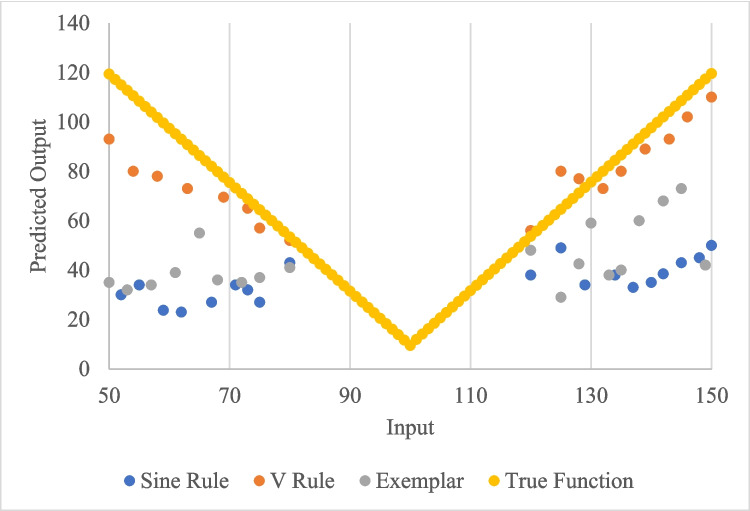
Extrapolation patterns of V rule learners, Sine rule learners, and Exemplar learners in comparison to the true function

Figure 5 presents the extrapolation pattern for V rule learners, sine rule learners, and exemplar learners in comparison to the true function. As can be seen, the V-rule learners’ extrapolation more closely matches the true function. The sine rule learners show a wave-like up and down pattern. The exemplar learners have a relatively flat extrapolation where the output values have the least variation.

#### Shape and letter-string categorisation

**Fig. 6:**
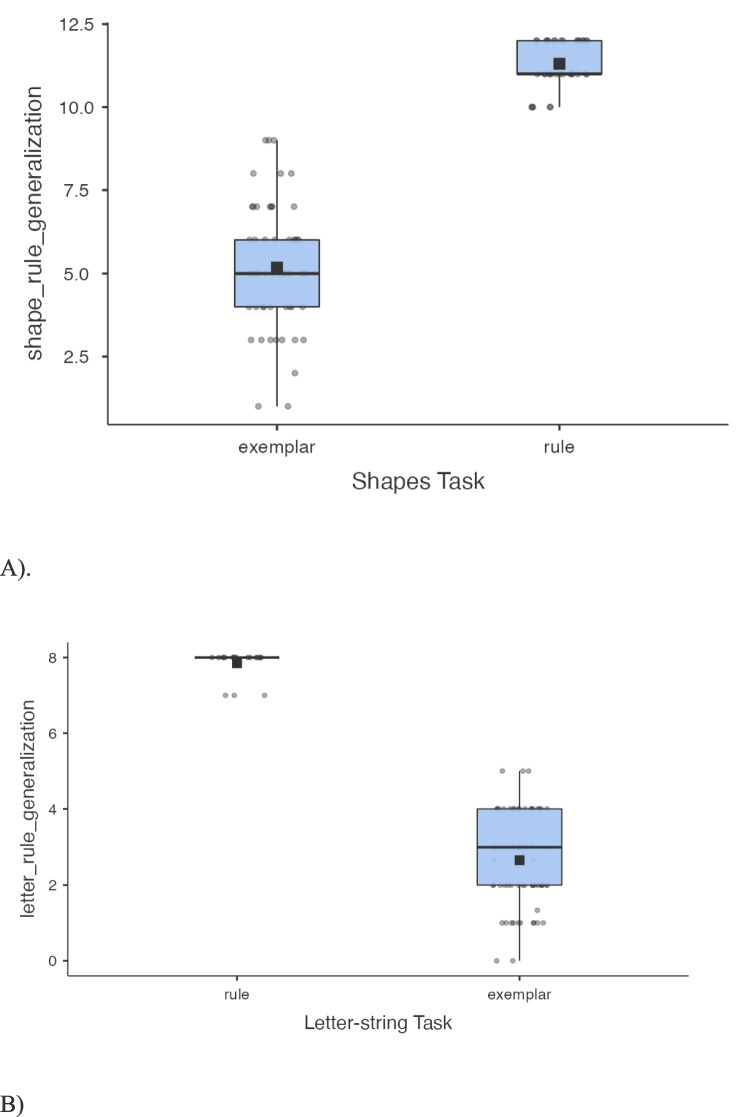
Box plots for rule and exemplar learners’ generalisation profiles for the shapes task (**A**) and the letter-string task (**B**)

Transfer scores for the shape task were calculated by adding the number of items correct on ambiguous transfer items to the number correct on novel transfer items. Since these items were diagnostic of rule abstraction, this composite measure reflected whether participants had discovered the rule during training (with higher scores indicating rule discovery). Similarly, transfer scores for the letter-string task were calculated by adding the number of items correct on ambiguous transfer items to the number correct on rule-favoured transfer items. For the shape and letter-string tasks, we dichotomised subjects into rule or exemplar learners based on their transfer scores. Participants were classified as rule learners on the shape and letter-string tasks if they had transfer scores of at least 10/12 and 7/8, respectively. Otherwise, participants were classified as exemplar learners. Only subjects with final training block performances ≥ 75% were included for analysis. See Table 1 for excluded participants in the shape and letter-string categorisation tasks. See Appendix Table 9 for means and standard deviations for training, unambiguous, and exemplar-favoured stimuli. See Fig. 6.

#### Patterning

**Fig. 7:**
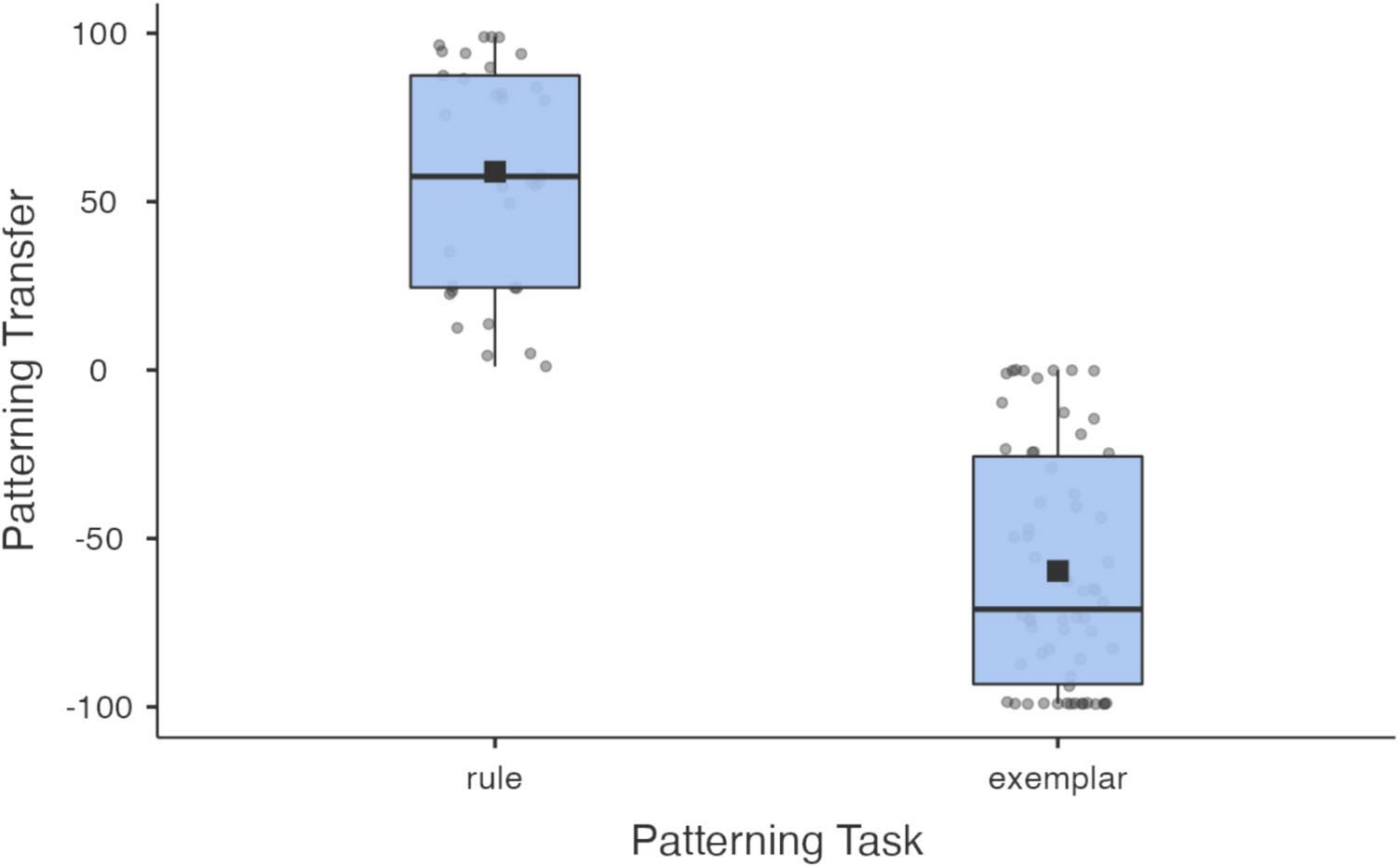
Box plot for rule and exemplar learners’ generalisation profiles for the patterning task

Transfer in this task was assessed by examining allergic reaction likelihood ratings on critical compound-cue test trials (*BananaAvocado? AvocadoBanana? BaconPeas?* and *PeasBacon?*). Recall that the rule flips how cues predict allergic reaction when they co-occur. If two cues individually predict an allergic reaction (such as *Banana* and *Avocado*), then when they co-occur, they predict no allergic reaction, and vice-versa. However, if one has not learned that rule, that *Banana* and *Avocado* co-occurring would elicit a prediction of an allergic reaction (given that they both make that predictions), and *Peas* and *Bacon* would elicit a prediction of no reaction. Transfer scores were created by subtracting average likelihood ratings between *BananaAvocado?* and *AvocadoBanana?* (which according to the rule, predict no reaction) from *BaconPeas?* And *PeasBacon?* (which according to the rule, predict a reaction). Thus, each subject had a transfer score from − 99 to 99, with positive scores indicating greater generalisation according to a rule, and negative scores indicating greater generalisation according to features. Subjects were then dichotomised based on this score: participants scoring > 0 were considered rule learners and ≤ 0, exemplar learners. Only those reaching criterion performance of 88.8% (32/36) in a training block were included for analysis (see Table 1 for excluded participants). See Fig. 7.

#### Picture-mapping

**Fig. 8:**
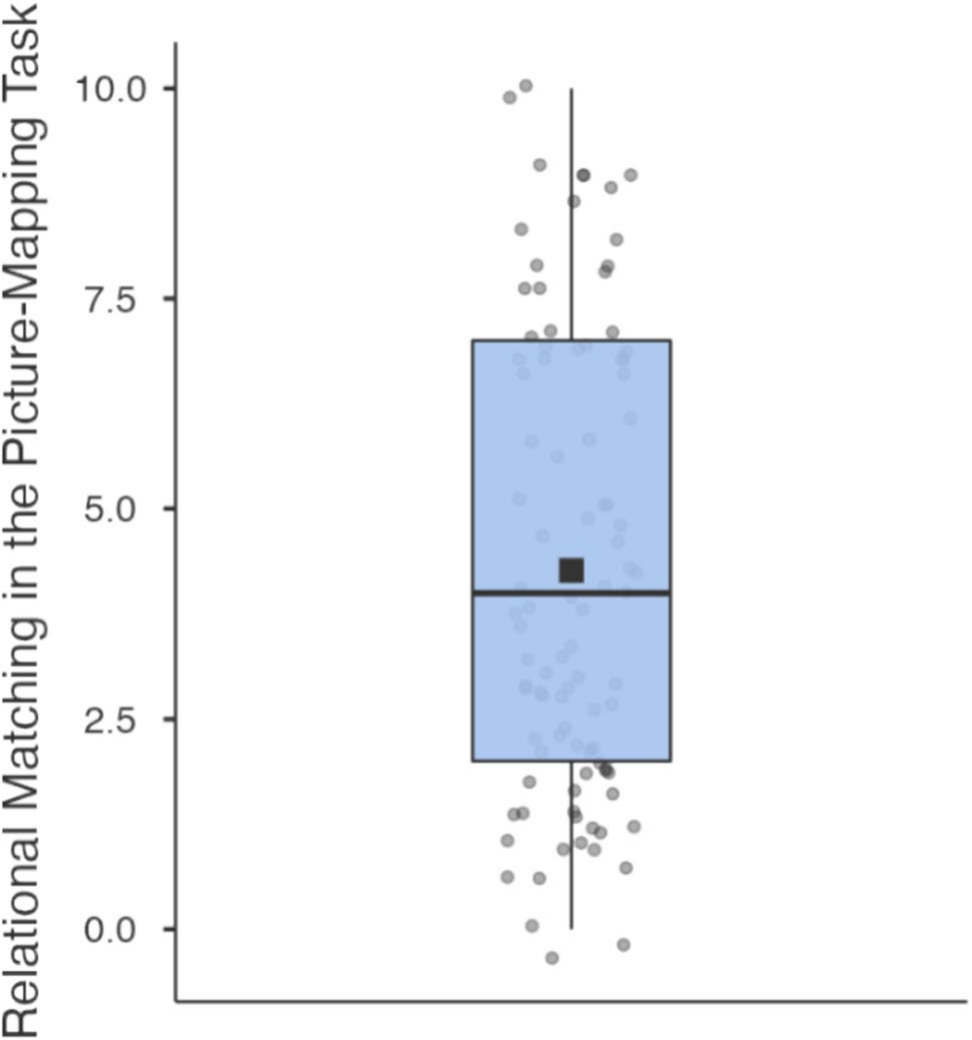
Box plot for rate of relational matching on the Picture-Mapping Task

Picture-mapping scores were calculated as the number of relational matches chosen across the ten scene pairs (see Table 1 for means and standard deviations; see Fig. 8).

#### Working memory

**Fig. 9:**
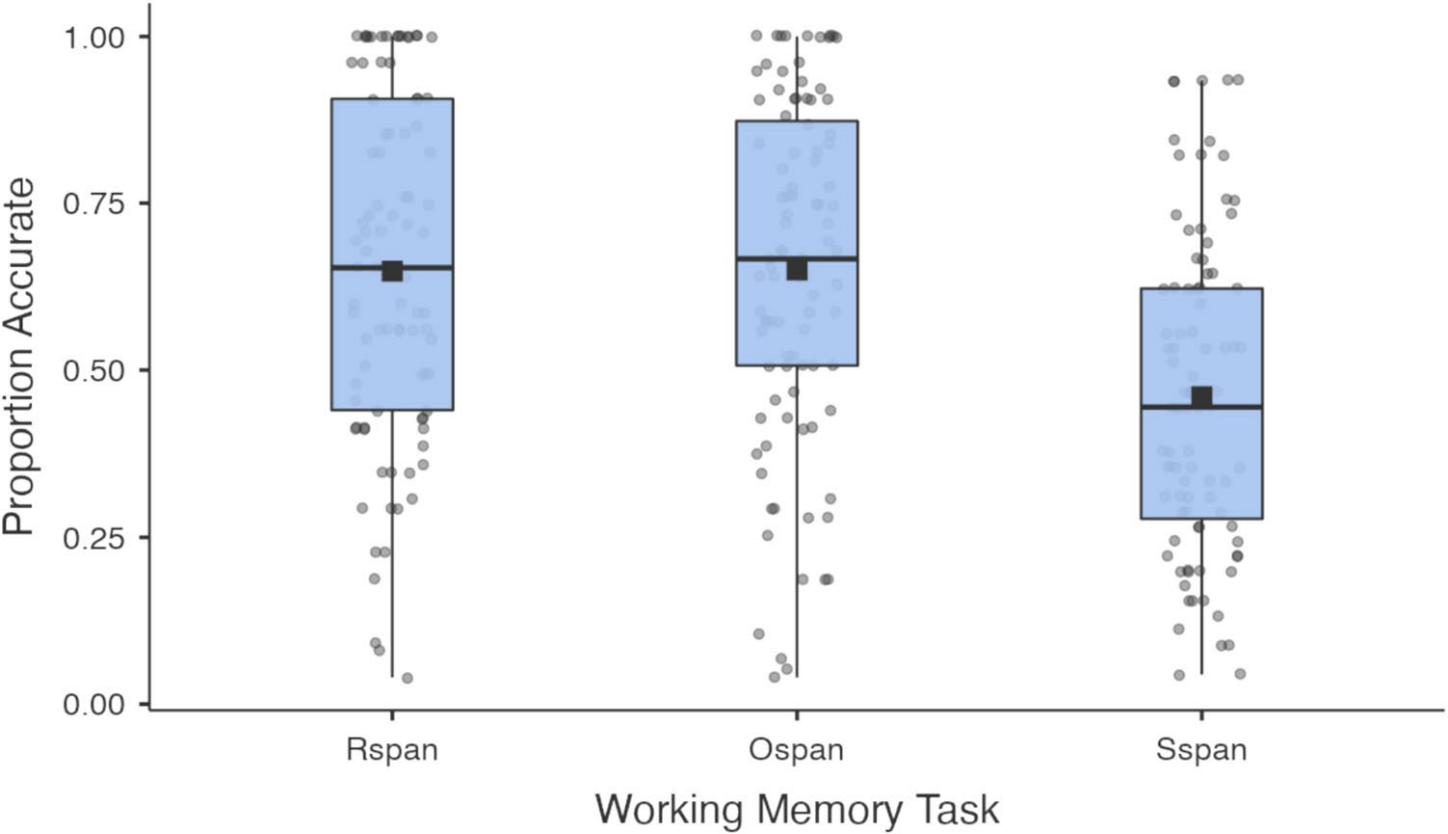
Box plots showing accuracy across three working memory tasks

Working memory scores for all three working memory tasks were calculated using the traditional ‘absolute’ scoring method (Engle et al., 1999; Turner & Engle, 1989), i.e., using the sum of all perfectly recalled sets. For example, if someone correctly recalls two letters in a set size of two, three letters in a set size of three, and three letters in a set size of four, their score is five (i.e., 2 + 3 + 0). As standard (Engle et al., 1999), subjects whose alternate task accuracy fell below 85% were excluded from analysis. Subjects with final scores of zero were also excluded as they had likely not seriously attempted the task (see Table 1 and Fig. 9).

### Descriptives

See Table 1 for frequencies and descriptives of the eight tasks used.

### Principal components analysis

A principal components analysis was conducted as the next step in answering our primary research questions concerning the stability of rule or exemplar-based learning strategies. We predicted the PCA would reduce the task set into two coherent and distinct dimensions of shared performance, corresponding to learning strategy and working memory capacity. In addition, this analysis tested whether there is any relationship between learning strategy and working memory capacity, even if they are reduced to two distinct components.

Relevant correlations between all measures were calculated and used to create a correlation matrix (see Table 2) that we analysed in FACTOR. Missing data were handled with pairwise exclusion. Principal components analysis (PCA) was performed with promax rotation. Three eigenvalues were greater than one; however, one of these was only just greater (1.04) and a Scree test (Cattell, 1966) suggested a two-component structure. Therefore, we specified FACTOR to extract two components (see Table 3 for component loadings and communalities). These two factors cumulatively accounted for 48% of the observed variance. Since the patterning task had poor communality (0.18), we removed this measure and re-estimated the solution. In this second analysis, two eigenvalues were greater than one, and a Scree test suggested two components. We therefore extracted two components (see Table 4).

**Table 2:** Correlation matrix for rule-versus-exemplar and working memory task scores

Variable	1	2	3	4	5	6	7	8
1. Function-learning	–							
2. Shape categorisation	0.401**	–						
3. Letter-string categorisation	0.317*	0.248**	–					
4. Patterning	0.240	0.220	−0.061	–				
5. Picture-mapping	0.307**	0.264*	0.100	0.048	–			
6. O-span	0.049	0.046	−0.037	0.141	0.053	–		
7. R-span	−0.009	0.066	−0.053	0.048	0.007	0.804**	–	
8. S-span	0.070	0.150	0.028	0.111	−0.008	0.243*	0.182	–

Tasks 1–4 resulted in dichotomous outcomes and 5–8, continuous outcomes. Thus, correlations here include tetrachoric, point-biserial, and Pearson. For tasks 1–4, exemplar learners were coded as ‘0’ and rule learners as ‘1’. * indicates p < 0.05; **, p < 0.01.

**Table 3:** Loadings and communalities from initial principal components analysis

	*Component loadings*		*h*^*2*^
	1	2	
Function-learning	−0.002	0.793*	0.63
Shape categorisation	0.081	0.739*	0.56
Letter-string categorisation	−0.144	0.536*	0.30
Patterning	0.212	0.359*	0.18
Picture-mapping	−0.005	0.549*	0.30
O-span	0.927*	−0.007	0.86
R-span	0.908*	−0.068	0.83
S-span	0.426*	0.158	0.21

Salient loadings (≥ 0.3) are indicated with *. h^2^ = communality.

**Table 4:** Loadings and communalities from second principal components analysis (patterning task removed)

	Component loadings		h^2^
	1	2	
Function-learning	0.014	0.782*	0.61
Shape categorisation	0.098	0.730*	0.55
Letter-string categorisation	−0.100	0.591*	0.36
Picture-mapping	0.024	0.574*	0.33
O-span	0.931*	−0.014	0.87
R-span	0.919*	−0.059	0.85
S-span	0.429*	0.147	0.21

Tables 3 and 4 clearly shows that the battery of tasks loaded onto two components as predicted. The first component we labelled ‘working memory’ and was defined by the three working memory measures. The second we labelled ‘strategy’ and was defined by all five (Table 3) and the remaining four of five (Table 4) original rule-versus-exemplar measures: function-learning, shape and letter-string categorisation, and picture-mapping. In the first solution, the patterning task also loaded on this component. These ‘working memory’ and ‘strategy’ components cumulatively accounted for 54% of the observed variance (28.3% and 25.6%, respectively) and were uncorrelated (r = 0.029).

**Fig. 10:**
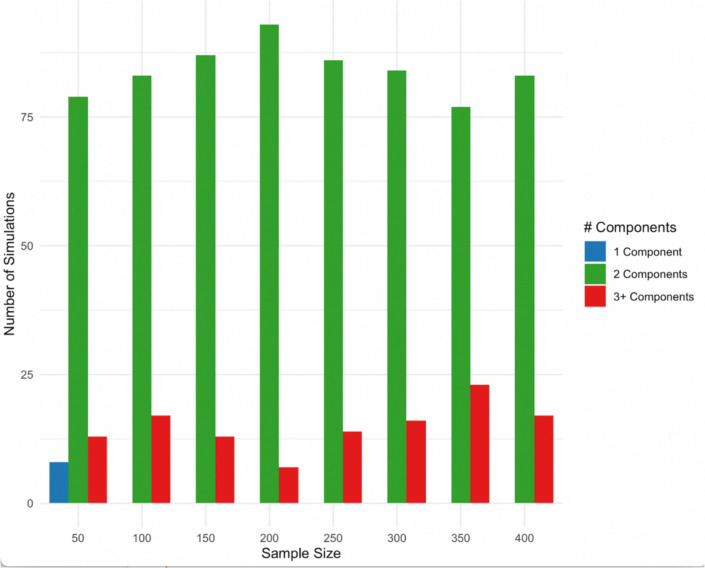
Parallel analysis results: Recommended number of components, based on 100 simulations per sample size

Next, we conducted Monte Carlo simulations to evaluate the stability of a two-component PCA solution. To do so, we generated multivariate normal data using the observed sample covariance matrix of eight variables, treating it as a proxy for the underlying population structure. This approach allows us to assess the internal replicability of our observed PCA result under repeated sampling conditions (which includes sampling error). Using the generated data sets, we applied parallel analysis to determine the optimal number of components. Parallel analysis compares observed eigenvalues to those expected from random data, retaining components whose eigenvalues exceed those from the simulated null model. An aim of parallel analysis is to determine whether an observed structure is likely to be a sample-specific artifact (Fabrigar et al., 1999; Hayton et al., 2004; Preacher & MacCallum, 2002; Zwick & Velicer, 1986). Parallel analysis has repeatedly been shown to outperform the more commonly used scree test and eigenvalue > 1 rule (which we used above), particularly in parallel analysis’ ability to avoid overfactoring (see above citations). That is, this analysis is more conservative. We conducted these analyses using the dplyr package for data manipulation (Wickham et al., 2023); multivariate normal data were generated using MASS (Ripley, 2002); parallel analysis with the psych package (Revell, 2023) and Fig. 10 was generated with ggplot2 (Wickham, 2016).

For each of a range of sample sizes (from 50 to 400, in increments of 50), we generated 100 datasets and applied parallel analysis. Across all sample sizes, parallel analysis consistently favoured a two-component solution, selecting it in over 75% of simulations at each level – suggesting the two-factor structure is robust across sampling variability. See Fig. 10.

Overall, our results show convergence for learning strategy measures, and convergence for working memory measures, respectively. That is, the learning strategy measures were correlated amongst each other sufficiently to load on one component, and likewise, the working memory tasks were so for a distinct component.

We note that one potential issue with the component-solution could have been that S-span was not as highly correlated with R-Span (*r* = 0.18) and O-span (*r* = 0.24) as they were with each other (*r* = 0.80), nor in the typical range of past work (0.55-0.73). In addition, S-span was the lowest performing of the three. The R-Span and O-Span tasks are superficially more similar to each other than either is to the S-span, but this did not prevent previous research from finding high correlations among all three. It is unclear why we got different results than past research, but we re-conducted the PCA without S-span to ensure this low correlation did not bias the solution. The two-component solution was maintained as before, and so we do not discuss it further.

### Confirmatory factor analysis

Next, we conducted CFA to test whether a two-factor latent structure could account for the pattern produced by the PCA, thereby providing stronger evidence for the presence of two dissociable constructs. We compared three nested models: (1) an independence model (0 factors), (2) a single-factor model, and (3) a theoretically motivated two-factor model aligned with the PCA structure. That is, we assessed how well a model with two latent factors, with the working memory tasks assigned to the first factor, and the learning strategy tasks assigned to the second fit the data using the lavaan R package (Rosseel, 2012). Model fit was assessed using the Comparative Fit Index (CFI), Root Mean Square Error of Approximation (RMSEA), Akaike Information Criterion (AIC), and Bayesian Information Criterion (BIC). For model comparison, the higher the CFI the better, and CFI equal to or above 0.95 is considered ‘excellent’. For AIC, BIC, and RMSEA, the lower the better, and for RMSEA, below 0.05 is considered a ‘close fit’.

Table 5 shows that the two-factor model aligned with the PCA results has the best fit across all four metrics. These metrics indicate that the two-factor structure provides an excellent and parsimonious account of the observed covariance patterns, while simpler models fail to adequately capture the data structure.

**Table 5:** Confirmatory factor analysis comparing three nested latent structure models

Model	CFI	AIC	BIC	RMSEA	Interpretation
0-factor	0.000	2187.45	2207.96	0.226	Very poor fit
1-factor	0.285	2156.26	2197.29	0.226	Still very poor fit
2-factor	1.000	2054.27	2097.87	0.000	Excellent fit

Thus, across the two-step analysis strategy of PCA and FCA, the data are consistent with both our first and second hypotheses – that there are distinct latent variables for learning strategy and for working memory. However, we note we failed to find evidence to support our third hypothesis, that there would be a positive relationship between rule learning and working memory.

#### Self-reported strategy and rule explication

The above analyses measured learning strategy through performance on generalisation tests. Here, we present exploratory analyses that examined relationships among self-reported learning strategies. These analyses aimed to shed light on the nature of the learning strategy construct by examining how available they are to learners’ direct introspection. Table 6 shows descriptives and Pearson correlations from the self-reported strategy Likerts. Self-reported strategy correlated significantly for shape and letter-string tasks, but not for those with the patterning task. This pattern corroborates the performance patterns where there is stability across the categorisation tasks but less connection with the patterning task.

**Table 6:** Descriptives and Pearson correlations for self-reported strategy Likerts

Measure	Descriptives		Correlation		
	Mean (N)	SD	1	2	3
1. Shapes self-report	5.23 (82)	2.14	–		
2. Letter-strings self-report	3.9 (80)	2.38	0.304**	–	
3. Patterning self-report	3.52 (86)	2.12	−0.072	0.109	–

** *p* < 0.01

Rule explication was assessed for shape, letter-string, and patterning tasks. For shapes, 49/82 (60.0%) subjects could partially or fully explicate the rule. For letters, 22/80 (27.5%) could explicate the rule. For patterning, 41/86 (47.7%) could explicate the rule.

### ‘Pure strategy’ self-report

The pure strategy measured was added to assess the distinction between initial attempts to learn the rule, and self-reported success of doing so. Data from the additional ‘pure strategy’ self-report was collected from 52 participants. Table 7 lists frequencies for subject responses and shows a large discrepancy between the number of subjects looking for a rule and those that found one. This finding is important because it adds context to findings, such as those in this paper, that uses performance to assess learning strategy. We found that many subjects (e.g., 38.6% in the shapes task) attempted to find a rule but could not. Also some subjects were not looking for a rule but found one. These findings suggest that the strategy used, as assessed by behavioural measures of generalisation may not line up with the conscious strategies with which learners start tasks.

**Table 7:** Frequencies for participant responses on ‘pure strategy’ self-report question

Measure	N	‘I was trying to find a rule, and did find a rule’	‘I was trying to find a rule, but did not find a rule’	‘I wasn’t trying to find a rule, but more-or-less accidentally discovered one’	‘I wasn’t trying to find a rule, and didn’t find a rule’	‘Not sure/none of these’	% rule attempters	% explicating rule
Shapes	44	22 (50.0%)	17 (38.6%)	0 (0.0%)	3 (6.8%)	2 (4.5%)	39 (88.6%)	24 (54.5%)
Letter-strings	44	12 (27.3%)	19 (43.2%)	2 (4.5%)	10 (22.7%)	1 (2.3%)	31 (70.5%)	14 (31.8%)
Patterning	47	13 (27.7%)	17 (36.2%)	6 (12.8%)	9 (19.1%)	2 (4.3%)	30 (63.8%)	23 (48.9%)

‘% rule attempters’ is the sum of the first two response options (i.e. I was trying to find a rule, and did find a rule plus I was trying to find a rule, but did not find a rule).

Table 8 shows correlations for the ‘pure strategy’ question. Here, only the shape and patterning tasks correlated significantly (0.78). These results differ from those found using the Likerts to measure self-reported strategy (i.e., Table 6) and the behavioural data that did not find strong connections between patterning tasks and the category learning tasks. This suggests that the patterning task may differ from the other learning tasks in how the rule is discovered, rather than eliciting an initial different approach.

**Table 8:** Correlation matrix for additional ‘pure strategy’ question

Variable	Correlations		
	1	2	3
1. Shapes rule attempt	–		
2. Letter-strings rule attempt	0.178	–	
3. Patterning rule attempt	0.783**	0.283	–

### Supplementary analysis

As a supplementary analysis, correlations were calculated for all relevant outcome variables (see Appendix Table 10). We note that generally, self-reports of learning strategy were significantly correlated with learning strategy task performance and not significantly correlated with working memory capacity.

Correlations here are tetrachoric. ‘Rule attempters’ (those who tried to find a rule, irrespective of finding one) were coded as ‘1’ and those responding all other options (tried to find a rule but did not, did not try to find a rule and did, did not try to find a rule and did not) were coded as ‘0’. ** indicates *p* < 0.01.

## Discussion

Our goal was to measure whether using rule- or exemplar-based learning strategies is a stable tendency that persists across tasks.

Using PCA on a set of five rule-versus-exemplar strategy tasks, we recovered a ‘strategy’ component, which indicates converging performance for rule-versus-exemplar tasks, providing evidence for strategy stability. We also recovered a working memory component from three working memory measures, providing divergent validity evidence for the strategy construct, and helping rule out that converging performance was due to an extraneous factor like motivation. We next tested whether the covariation pattern was best modelled as arising from two underlying latent factors. The confirmatory factor analysis showed excellent fit for a two-factor latent structure, exceeding the models with one or zero factors, which both showed poor fit.

This two-step analytic strategic provides strong evidence that working memory and learning strategy are distinct psychological constructs, such that exemplar learners are not rule-learners simply because they do not have sufficient working memory resources to search for and discover abstract rules. Using the methods from personality and intelligence research analysing latent variables from a battery of tasks, the current paper builds on prior work which provided the initial evidence for this dissociation between learning strategy and cognitive capacity (working memory and fluid intelligence) with just a pair of tasks (Goldwater et al., 2018).

Contrary to our third hypothesis, the strategy component was not just dissociated from working memory capacity, it was not related to the working memory component at all. Prior studies indicated a relationship between individual tasks from the rule-learning set and working memory (Gray & Holyoak, 2020; McDaniel et al., 2014; Wills et al., 2011). However, we did not find evidence for this. For example, Gray and Holyoak (2020) included the picture-mapping task and a measure of symmetry span. They found a significant correlation of *r* = 0.22. We found a non-significant correlation of *r* =  − 0.08. Although we are quite confident that working memory and learning strategy form distinct components, we are less confident that the two have no relationship at all.

Building on the above findings of converging performance for rule-versus-exemplar tasks, we also found convergence for self-reported strategy measures. There was a significant positive correlation in self-reported strategy between the shape and letter-string categorisation tasks. Further, when using a novel ‘pure strategy’ question, we found convergence for the shapes and patterning task. Likewise, none of the self-report measures showed significant correlations with working memory. Thus, we provide an array of new evidence that learning strategies are consistent across tasks and clearly distinct from working memory capacity.

However, the self-report data also raised more questions about the nature of learning strategies. The distinction in findings between attempted learning strategy (which saw a connection between categorisation and patterning tasks) and the learning strategy shown by the behavioural data (where the patterning task did not show as strong a connection to categorisation tasks) raised questions specifically about what determines successful strategy use, and how failure may lead to a shift in strategies. Likewise, past research has shown how task performance can prompt strategy-switches (Gouravajhala et al., 2020). Perhaps contrasting the nature of rule learning across these tasks can be illuminating. The patterning task is distinct from the categorisation tasks because encoding every item’s predictive value is a prerequisite to rule application for the patterning task, while for categorisation tasks, applying the rule does not require any exemplar memory across trials. Future research is needed to determine what sets this rule-discovery process apart from category and function-learning tasks (see Don et al., 2020).

These findings that working memory has no relationship to rule learning, prompts at least one more big open question. If learning strategy differences are not based on working memory differences, why do people differ in their learning strategies? We do not have an answer. First, we note a limitation of our study is that the only cognitive capacity we tested was working memory. The literature suggested working memory as the best candidate individual cognitive difference to be associated with learning strategy, but this does not rule out that other differences, such as cognitive flexibility, metacognition, inhibitory control, or planning capabilities would not show a strong relationship with rule-learning strategy. More thorough investigation of the relationships between learning strategy and these other cognitive capacities would be needed to fully support the claim that learning strategy is a unique cognitive trait, arising from a unique latent variable. Currently, we have evidence to say that learning strategy and working memory capacity are distinct cognitive constructs.

In addition to conducting a similar study to the current research swapping out a working memory battery for another cognitive capacity, we recommend another research approach. Given the evidence of connections between learning strategy to undergraduate performance (McDaniel et al., 2018, 2022) and self-reported study habits (Goldwater et al., 2018) it seems these differences are established by the time students enter university. This suggests research needs to dig into primary and secondary school to understand their developmental origins.

To aid in researching the origins of the strategies, the cognitive processes underlying the different strategies need to be better characterized. As argued in the *Introduction*, it appears that a rule-strategy is related to a tendency to compare multiple stimulus elements. We found further evidence for this with the picture-mapping tasks loading on the learning strategy component. This task measures a preference for finding relational correspondences, which is best fostered by engaging in analogical comparison (Markman & Gentner, 1993). Recent work highlights that from the second year of life, children vary in how much their conversations with parents use language that supports analogical comparison and relational cognition (Frausel et al., 2020, 2021). It is possible that the origins of focal orientations towards abstract relations will show roots in these early parent–child interactions. Though we note at this point, this is purely a speculative connection.

Likewise, the cognitive processes underlying exemplar strategies need further illumination. An anonymous reviewer asked if the strategy relies on memorisation, or a similarity with attention process using item-based category representations? The current research suggests that exemplar strategies are associated with feature-based generalisation (e.g., generalisation in the function task is limited to the range of learned instances), but it cannot further identify underlying processes to distinguish among the many processes that support feature-based generalisation offered in the literature. Insights could be gained however by characterising the *cognitive affordances* of the tasks in the current battery and related prior research.

The two tasks from Little and McDaniel (2015) repeatedly exposed subjects to the same 12 exemplars throughout the learning phase, encouraging rehearsal. Likewise, the function learning task presented 20 input–output pairs ten times each during the training period. The patterning task requires remembering each specific association between item and outcome. Those exemplar strategies may all involve memorisation in the typical sense. However, the picture-mapping task does not require encoding anything into memory as each trial is independent from the next. So, tapping into the exemplar learning strategy does not require a task that requires learning and memory, just attention. In addition, the task discussed from Goldwater et al. (2018) randomly generates exemplars (within the constraints of the categories), and items are not repeated across learning trials, and so memorising specific exemplars seems unlikely. Thus, all the exemplar learning patterns show attention to intrinsic features of the stimuli, but the degree to which the tasks afford precise memory of every exemplar varies. So, it does not seem that memorisation is essential to the exemplar focal orientation, but memorisation does appear to be deployed by exemplar learners when the task affords or requires it.

Characterising learning tasks’ cognitive affordances is the flip side to identifying the focal orientations of learners entering the task. As another anonymous reviewer suggested, important to moving this area of research forward will be identifying how people perceive the cognitive affordances of tasks, and how that can change over time (Gouravajhala et al., 2020). This will support an experimental approach wherein task factors are manipulated to change how participants perceive the affordances.

Because the rule-and exemplar processes are potentially available to all learners, ultimately a process model that shows how they co-exist in the same mind will be necessary. There are already process models of categorisation that show how rule and exemplar representations emerge from the same learning process (in a cluster model by Jones & Love, 2006, and in a hierarchical Bayesian model by Lee & Vanpaemel, 2008). Here, based on learning-task affordances and structure, more local rules (corresponding to specific exemplars) or more global rules may be abstracted. These kinds of approaches may prove useful to explaining why people differ in forming these representations and how they switch between strategies, especially if modified to discriminate between relational and feature-based rules.

Understanding the relationships between task affordances and learner strategies raises a third important line of future research – how to foster successful learning strategies when students are regulating their own learning. Many studies show how to scaffold elaborative processes of self-explanation and comparison in formal education (see Bisra et al., 2018, and Alfieri, Nokes-Malach, & Shunn, 2013, respectively,) but it seems that once these scaffolds are removed, students resort to their default focal orientation. The work by McDaniel and colleagues (2018; 2022) and Zepeda and Nokes-Malach (2021) shows that rule-based and elaborative strategies are advantageous for undergraduate STEM performance, and so work is needed to identify how to support their spontaneous use. There is already work that successfully trains the self-regulated use of high quality memorisation strategies (such as retrieval practice), so it seems plausible to support rule-based learning strategies as well (McDaniel & Einstein, 2020).

The goal of course is not to eliminate strategies that support memorisation, but to ensure that students do more than just memorize to foster knowledge transfer to novel contexts. We hope these suggestions point the field towards a unification of formal model driven experimental work on learning, and applied work in education.

Last, we note a few more limitations of the current study. Firstly, a significant proportion of participants’ function-learning data were excluded from analysis due to not meeting the learning criterion. Further, even though we reached the minimum required sample size to uncover two latent variables from eight tasks (Mundfrom et al. 2005), and the two-factor latent structure model fit the data well, ideally the sample would be larger. This was our motivation for conducting the parallel analysis based in Monte Carlo simulations, a more conservative analysis than more common approaches to component or factor selection. This analysis favoured the two-component structure reliably across a large range of sample sizes. Of course, Monte Carlo simulations are not immune to bias, they necessarily reflect the structure of the data collected, but they build in variation due to sampling error with the aim to identify results that are artifacts of the sample. If the sample data poorly reflect the true population beyond this, then like any other statistical method, parallel analysis may lead to the wrong conclusion.

Perhaps another limitation is that unlike some work just mentioned above, the current work did not measure learning strategies used in education. We did so because this was not a university student sample (it was an online sample). Thus, the current sample limitations suggest the need to further extend the approach to a larger sample of students across educational levels (tertiary and earlier) to understand the stability and origin of learning strategies. Finally, an anonymous reviewer questioned whether the stability across tasks was a priming effect – perhaps participants simply continued using whichever strategy was used first. Although we cannot easily rule this explanation out in the current dataset, given the connection to education performance and self-report, this priming effect interpretation seems less parsimonious with the literature as a whole.

## Conclusion

Here we provided further evidence for learning strategy (rule or exemplar based) as a stable focal orientation, persisting across conceptually disparate tasks. Also, despite mixed prior findings, we did not find evidence of a relationship between learning strategy and working memory. We consider this project an advance towards the goal of connecting theory-driven experimental work on concept learning and transfer with individual differences research in cognition.

## Appendix I Stimuli for experimental tasks

**Shape Task Items**

**Learning items**

**Example original generalization items**

**Novel transfer items used in the shape categorisation task.**

**Items for Letter String Task**

## Appendix II

**Table 9:** Means and SDs for variables ancillary to main analyses

Task	Mean	SD
Function-learning (*N*=71)		
Final training block mean error	5.56	3.69
Interpolation mean error	7.21	3.90
Shape categorisation (*N*=82)		
Training items	3.62	0.90
Ambiguous	2.21	1.51
Unambiguous	3.45	0.92
Novel	5.66	2.26
Letter-string categorisation (*N*=80)		
Training items	3.9	0.33
Ambiguous	1.20	1.64
Rule-favoured	2.68	1.16
Exemplar-favoured	3.15	0.95

## Appendix III

**Table 10:** Correlation matrix of all dependent variables in the current study

	1	2	3	4	5	6	7	8	9	10	11	12	13	14	15	16	17
1. Function-learning rule or exemplar	–																
2. Shapes rule or exemplar	**0.401****	–															
3. Shapes self-report	0.207	0.574**	–														
4. Shapes rule attempt	–0.319	0.502**	0.156	–													
5. Shapesrule explication	0.204	0.925**	0.616**	0.666**	–												
6. Letter-strings rule or exemplar	**0.317***	**0.248***	0.304**	0.131	0.387**	–											
7. Letter-strings self-report	0.107	0.101	*0.304***	0.184	0.153	0.672**	–										
8. Letter-strings rule attempt	–0.097	0.074	0.099	0.178	–0.100	0.552**	0.384*	–									
9. Letter-strings rule explication	0.428**	0.250*	0.289*	0.211	0.451**	0.993**	0.689**	0.412**	–								
10. Patterning rule or exemplar	**0.240**	**0.220**	0.011	0.023	0.340**	**-0.061**	–0.116	–0.522**	–0.137	–							
11. Patterning self-report	0.036	–0.041	–*0.072*	0.145	0.013	0.113	*0.109*	0.284	0.121	0.219*	–						
12. Patterning rule attempt	0.426*	0.475**	–0.029	0.783**	0.305	0.183	0.239	**0.283**	0.296	0.175	0.490**	–					
13. Patterning rule explication	0.248*	–0.120	–0.188	–0.060	0.204	0.000	–0.056	–0.734**	0.098	0.741**	0.326**	0.187	–				
14. Picture-mapping	**0.307****	**0.264***	0.127	–0.018	0.289**	**0.100**	0.105	0.071	0.121	**0.048**	0.012	0.052	0.094	–			
15. O-span	**0.049**	**0.046**	0.154	–0.191	0.078	**–0.037**	0.142	–0.162	0.005	**0.141**	0.298**	0.149	0.031	**0.053**	**–**		
16. R-span	**–0.009**	**0.066**	0.106	**–**0.201	0.125	**–0.053**	0.052	–0.214	**–**0.007	**0.048**	0.144	0.008	0.045	**0.007**	**0.804****	**–**	
17. S-span	**0.070**	**0.015**	0.291*	**–**0.208	0.183	**0.028**	0.004	–0.360*	0.008	**0.111**	0.012	**–0**.042	0.054	**–0.008**	**0.243***	**0.182**	–

*Note:* * indicates *p* < .05; **, *p* < .01. Values in bold indicate correlations presented in Table 2. Italicised values are from Table 5. Underlined values are from Table 7

## Declarations

### Open practices

The data are available at https://osf.io/acbmu/?view_only=6de1e020e61848a2b80e2fb1c06df7ad
